# True 3D Nanometrology: 3D-Probing with a Cantilever-Based Sensor

**DOI:** 10.3390/s22010314

**Published:** 2021-12-31

**Authors:** Jan Thiesler, Thomas Ahbe, Rainer Tutsch, Gaoliang Dai

**Affiliations:** 1 Physikalisch-Technische Bundesanstalt (PTB), Bundesallee 100, 38116 Braunschweig, Germany; thomas.ahbe@ptb.de; 2 IPROM, Technische Universität Braunschweig, Schleinitzstraße 20, 38106 Braunschweig, Germany; r.tutsch@tu-bs.de

**Keywords:** 3D nanometrology, atomic force microscopy (AFM), critical dimension AFM (CD-AFM), true 3D-AFM, 3D sensitivity, 3D-Nanoprobe

## Abstract

State of the art three-dimensional atomic force microscopes (3D-AFM) cannot measure three spatial dimensions separately from each other. A 3D-AFM-head with true 3D-probing capabilities is presented in this paper. It detects the so-called 3D-Nanoprobes CD-tip displacement with a differential interferometer and an optical lever. The 3D-Nanoprobe was specifically developed for tactile 3D-probing and is applied for critical dimension (CD) measurements. A calibrated 3D-Nanoprobe shows a selectivity ratio of 50:1 on average for each of the spatial directions *x*, *y*, and *z*. Typical stiffness values are $k_{x}\;=\;1.722\;\pm \;0.083$ N/m, $k_{y}\;=\;1.511\;\pm \;0.034$ N/m, and $k_{z}\;=\;1.64\;\pm \;0.16$ N/m resulting in a quasi-isotropic ratio of the stiffness of 1.1:0.9:1.0 in *x*:*y*:*z*, respectively. The probing repeatability of the developed true 3D-AFM shows a standard deviation of 0.18 nm, 0.31 nm, and 0.83 nm for *x*, *y*, and *z*, respectively. Two CD-line samples type IVPS100-PTB, which were perpendicularly mounted to each other, were used to test the performance of the developed true 3D-AFM: repeatability, long-term stability, pitch, and line edge roughness and linewidth roughness (LER/LWR), showing promising results.

## 1. Introduction

**Figure 1:**
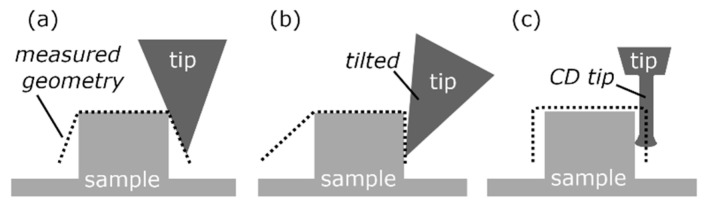
Measured (apparent) geometries without tip-deconvolution (dotted line) of the conventional atomic force microscope (AFM) (**a**), the tilting AFM (**b**), and the two-dimensional (2D)/critical dimension (CD) AFM (**c**) with the specialized cantilever with a CD tip.

The atomic force microscope (AFM), invented in 1985 [1], is probably the most used scanning probe microscope, and it measures surface topography non-destructively at a sub-nanometer resolution [2,3,4]. The AFM’s probe is most often a small beam of rectangular or triangular shape and is called a cantilever. The cantilever is deflected under the close- and far-range acting forces of the interacting interatomic potentials between its tip and the sample surface. Several detection mechanisms such as inductive, capacitive, piezo resistive, and/or interferometric sensors are known, but in most cases an optical lever [2,3] is used. Hence, the bending deflection of the cantilever is detected, which makes the conventional AFM-head a one-dimensional (1D) sensor [5]. With a scanning motion between the sample and the cantilever while probing, the contour of the surfaces is traced. Conventional AFMs are applied in research and industry and are designed to measure relatively flat surfaces often with oftentimes high resolutions down to the picometre range in the in vertical direction. They and are used in measurements tasks including those that examine step height, the pitch of gratings, trenches, and roughness. Figure 1a shows, the measured apparent contour of a sample, which is a convolution between the tip of the cantilever and the surface. One can see that the conventional AFM with a conventional tip has a no ability to probe steep sidewalls. The steepest measurable sidewall angle is limited to about half of the opening angle of the cone-shaped tip. To overcome this restriction, the tilting-AFM in Figure 1b was invented. It can tilt the cantilever and measure the contour of the sample [5,6]. By matching multiple measurements of the same region of the sample with varying tilting angles, the complete contour of the sample is extracted.

Figure 1c shows that just one measurement is needed for the so-called critical dimension (CD)/two-dimensional (2D) AFM [7]. This CD AFM is specifically designed to measure vertical side walls and is widely used for critical dimension measurements (such as features width, edge profiles, sidewall angles, corner rounding, contours, line edge roughness) and slightly undercut sidewalls for quality assurance in lithography processes and research [8,9]. The probe used in this CD AFM is a cantilever with a conical tip and a cylindrical tip apex. This cylindrical tip apex (diameter 850 nm to 15 nm) is flared at the very end and is known as a CD tip. It has a length-to-diameter ratio of about 6:1 and allows probing in the horizontal and vertical direction by dual-axis tip control. The cantilever bending and torsion, are frequently detected via a dual-optical lever, which makes this CD AFM head a 2D sensor [5,7,8]. The CD AFM and tilting-AFM are oftentimes called three-dimensional (3D) AFMs but have no true 3D-sensing capabilities, mainly because of limitations in cantilever mechanics, which are explained in [10]. Several approaches to, and developments in, true 3D-sensing or true 3D-AFMs have been made [8,9,10,11,12,13,14,15,16,17] with promising results. Unfortunately, most of the systems are still under development.

In this paper, a true 3D AFM with full 3D-probing capabilities is presented. Section 2 explains limitations of the commercial cantilever AFM probe and the developed cantilever-based 3D-Nanoprobe with a CD-tip. Moreover, the detection scheme and principle are explained. In Section 2.2 expected calibration coefficients are modeled in detail. The calibration coefficient of the cantilever’s probing curve in a conventional AFM for 1D measurement is replaced by a 3 × 3 matrix for the 3D-Nanoprobe. The setup that was built within this research project is described in Section 2.3. It detects the 3D-Nanoprobes deflection within the 3D-AFM-head by using an optical detection unit combined with an optical microscope for sample inspection. In Section 3, the 3D-Nanoprobes stiffness is calibrated via a reference spring method and is also tested for repetitive probing. Moreover, the 3D-Nanoprobe is applied for measurements of CD-structures, pitch, and roughness with very promising results. 

## 2. Materials and Methods

### 2.1. Design Concept and Detection Principle of the 3D-Nanoprobe

**Figure 2:**
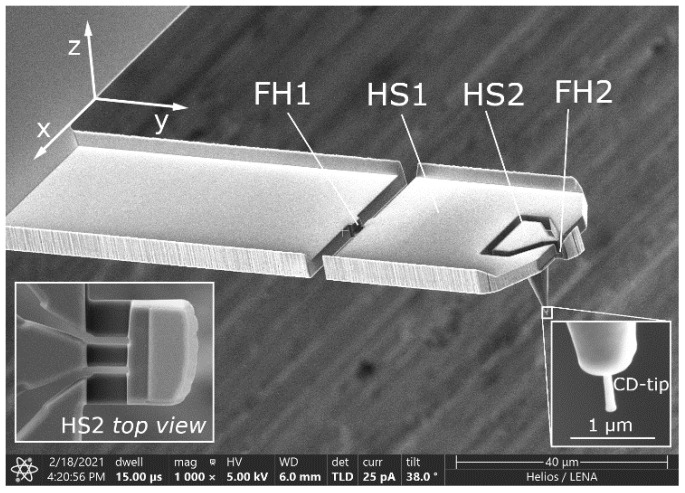
Scanning electron microscope image of a manufactured 3D-Nanoprobe with two flexure hinges (FH 1 and FH2) and two head sections (HS1 and HS2), the details show the CD tip with the flared tip apex and the structure of HS2.

The commercial CD-tip cantilever has several limitations for 3D probing, such as a crosstalk in the *yz*- and *zy*-directions, a strong anisotropy ratio in compliance and small detectable signal for slender and compliant CD tips, as detailed in [10]. The 3D-Nanoprobe [10] was designed to overcome these limitations, which might lead to shaft probing [18,19,20], tip breakage [21], or measurement errors. It is based on a rectangular CD tip cantilever and is manufactured with focused ion beam (FIB). Figure 2 shows the structure of the 3D-Nanoprobe with two head sections (HS1 and HS2) separated by two compliant flexure hinges (FH1 and FH2).

**Figure 3:**
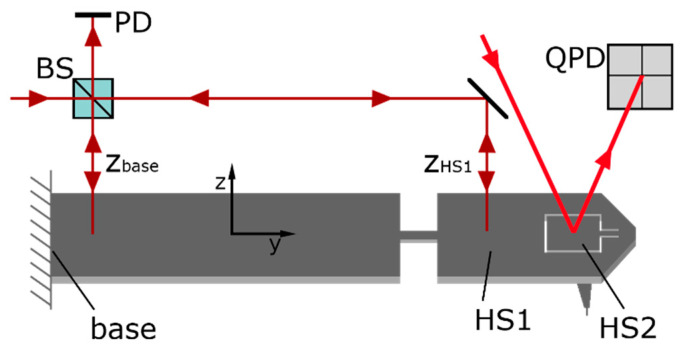
Abstract detection scheme of the 3D-Nanoprobe with a differential interferometer (beam splitter (BS), photodiode (PD)) to detect the displacement of HS1 z_HS1_ relative to the displacement of the base z_base_ in the *z*-direction and a dual optical lever to detect a change in the inclination angle of bending angle *α* and torsion angle *β* of HS2 on the segmented quadratic photodiode (QPD).

The first head section, HS1, is suspended by the first flexure hinge, FH1; and the second head section, HS2, is suspended by the second flexure hinge, FH2, which is connected to HS1. The tip cone is connected to HS2, and the tip apex is a flared CD tip. The 3D-Nanoprobe is optimized for the quasi-isotropic ratio of the stiffness for the *x*-, *y*-, and *z*-directions. It is also optimized for high sensitivity by reducing the overall stiffness and by adapting it to the CD tip’s mechanical properties along with the separated resonant frequencies and diminished crosstalk, as detailed in [10]. In this paper, the 3D-Nanoprobes tip displacement is detected via an optical detection unit consisting of an interferometer and an optical lever. The optical detection scheme of the 3D-Nanoprobe is shown in Figure 3. A laser beam is split into two beams by the beam splitter (BS). One beam is positioned on the base as a reference arm to reduce drift and environmental influences. The second beam is positioned on HS1 to measure the displacement in the *z*-direction forming a differential interferometer.

**Figure 4:**
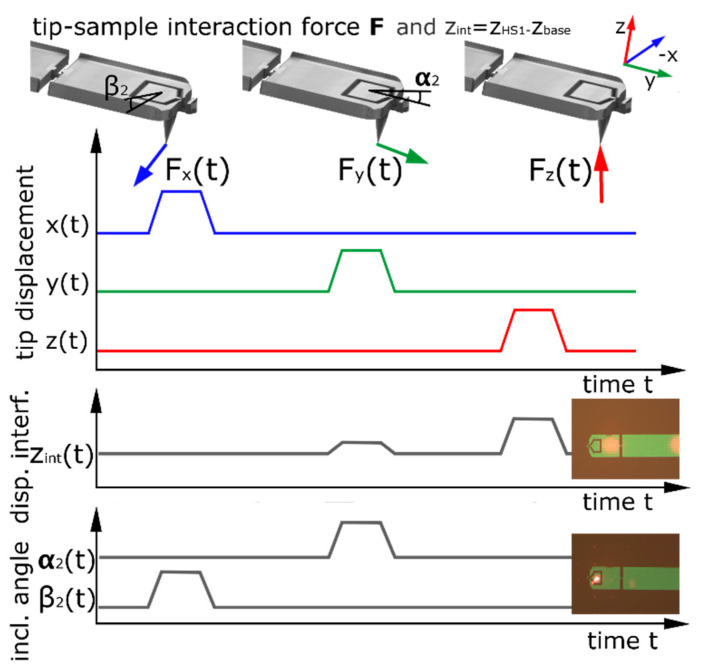
Detection principle of the tip–sample interaction of the 3D-Nanoprobe with the interferometer focused on HS1 and the dual-optical lever focused on HS2.

In general, the *z*-displacement can also be measured on HS2, which has three main advantages compared to HS1: the signal is larger (about two times larger displacement compared to HS1), the *z*-displacement shows no change in the inclination angle of HS2, as mentioned in [10], and there is only the tip between the sample and the measurement surface. The disadvantage of measuring on HS2 compared to HS1 is a larger displacement in the horizontal *x*-direction, a larger change in the inclination angle of the surface for horizontal forces, which might influence the collimation of the interferometer beam, and a smaller surface for aligning the beam. In practice, the interferometer spots are currently about 19 µm in diameter. Such a spot is too big for HS2; hence, the second beam is focused on HS1. The laser of the dual-optical lever is aimed at HS2 and reflected onto a quadratic photodiode (QPD) to detect the changing inclination angles $\beta_{2}$ and $\alpha_{2}$, in orthogonal directions. Figure 4 illustrates the principle of tip displacement and detected signals. The displacement on HS1 is biased by a crosstalk between z and y. With the assumption of a strong correlation between the horizontal CD-tip displacements and the inclination angles of HS1 and HS2, this crosstalk is proportional to the measured bending angles of HS2 and can therefore be compensated for. The change in inclination angle of HS2 will be zero for a specific optimized force direction at the 3D-Nanoprobes tip apex. This force direction denotes the *z*-direction, as indicated. With a calibration procedure, the three orthogonal displacements of the tip can be selectively detected.

This detection principle is used and explained in detail in the next subsection. In practice, wrong initial calibration coefficients might damage the 3D-Nanoprobes CD tip. Consequently, a detection model is built by deducing calibration coefficients from modelled sensitivity matrix elements.

### 2.2. Modelling of the 3D-Nanoprobes Detection Principle and Calibration Values

In this chapter, the detection principle of the AFM head is applied in theory to the optimized 3D-Nanoprobe. The measured signal in relation to the tip displacement in all three spatial directions is described by the sensitivity matrix. Hence, a first set of sensitivity matrix elements can be derived from the matrix of the inclination angles and the corresponding stiffness matrix. With the properties of the optical detection unit and the mechanics of the 3D-Nanoprobe, the sensitivity matrix is used to calculate an estimate of the calibration coefficients. Afterwards, the calibration procedure is explained, and this is then verified by an experiment.

#### 2.2.1. Modeling the Detection Principle of the 3D-Nanoprobes

The dual-optical lever is positioned on HS2 and detects its inclination angles. The force $\vec{F}$-dependent inclination angles of the dual-optical lever for the 3D-Nanoprobe are explained in [10] and can be described by a matrix as

(1) $$\left(\begin{array}{c} \alpha_{2}\\ \begin{array}{c} \beta_{2}\\ \gamma_{2} \end{array} \end{array}\right)=\left[\begin{array}{ccc} 0 & \alpha_{y2} & \alpha_{z2}\\ \beta_{x2} & 0 & 0\\ \gamma_{x2} & 0 & 0 \end{array}\right]\left(\begin{array}{c} F_{x}\\ F_{y}\\ F_{z} \end{array}\right)$$

with $\alpha_{y2}$ and $\alpha_{z2}$ as matrix coefficients of the bending inclination angle $\alpha_{2}$ of the tip–sample interaction forces in the *y*- and *z*-direction, respectively. The torsion inclination angle $\beta_{2}$ is deduced by the matrix coefficient $\beta_{x2}$ and the tip–sample interaction force in the *x*-direction. $\gamma_{2}\;$is the in-plane bending angle and cannot be detected by the dual-optical lever. 

In the setup, the 3D-Nanoprobe is tilted intentionally by an incident angle φ of 8° to ensure enough safety distance between the 3D-Nanoprobe’s clamping mechanics and the sample surface. This angle must be considered in the model for later calibration because it changes the matrices of the inclination angles and the compliances. Therefore, the matrix of inclination angles in Equation (1) is rotated around the *x*-axis by φ. The expression for the force-dependent detectable inclination angles $\alpha_{2}$ and $\beta_{2}$, at HS2 changes to

(2) $$\left(\begin{array}{c} \alpha_{2}\\ \beta_{2} \end{array}\right)=\left[\begin{array}{ccc} 0 & \alpha_{y2}\cos\left(\varphi \right)-\alpha_{z2}\sin\left(\varphi \right) & \alpha_{y2}\cos\left(\varphi \right)+\alpha_{z2}\sin\left(\varphi \right)\\ \beta_{x2} & 0 & 0 \end{array}\right]\left(\begin{array}{c} F_{x}\\ F_{y}\\ F_{z} \end{array}\right)$$

The common compliance matrix C of a cantilever-based probe is given in [10] by

(3) $$C=\left[\begin{array}{ccc} c_{xx} & 0 & 0\\ 0 & c_{yz} & c_{yz}\\ 0 & c_{zy} & c_{zz} \end{array}\right]\;$$

It is rotated by φ and inverted to get the stiffness matrix K, $C^{-1}\;=\;K$. Implementing this in the general expression $K\;*\;\vec{d}\;=$ $\vec{F}$ leads to

(4a) $$\left[\begin{array}{ccc} \frac{1}{c_{xx}} & 0 & 0\\ 0 & \frac{c_{zy}\sin\left(\varphi \right)+c_{zz}\cos\left(\varphi \right)}{c_{det}} & \frac{c_{yz}\sin\left(\varphi \right)+c_{yz}\cos\left(\varphi \right)}{c_{det}}\\ 0 & \frac{c_{zy}\cos\left(\varphi \right)-c_{zz}\sin\left(\varphi \right)}{c_{det}} & \frac{c_{yz}\cos\left(\varphi \right)-c_{yz}\sin\left(\varphi \right)}{c_{det}} \end{array}\right]\left(\begin{array}{c} x\\ \begin{array}{c} y\\ z \end{array} \end{array}\right)=\left(\begin{array}{c} F_{x}\\ F_{y}\\ F_{z} \end{array}\right)$$

With the determinate of the compliance

(4b) $$\begin{array}{c} c_{det}\;=\;(c_{yz}\cos\left(\varphi \right)-c_{yz}\sin\left(\varphi \right))\;*\;\left(c_{zy}\sin\left(\varphi \right)+c_{zz}\cos\left(\varphi \right)\right)-\left(c_{yz}\sin\left(\varphi \right)+c_{yz}\cos\left(\varphi \right)\right)\\ \;*\;\left(c_{zy}\cos\left(\varphi \right)-c_{zz}\sin\left(\varphi \right)\right) \end{array}$$

and the tip apex displacement $\vec{d}\;=\;\left(\begin{array}{c} x\\ \begin{array}{c} y\\ z \end{array} \end{array}\right)\;$.

The stiffness matrix multiplied by the displacement of the tip expresses the forces acting on the tip apex. The force vector in Equation (4a) is implemented in Equation (2). Equation (5) describes the changes in inclination angles for bending$\;\alpha_{2}$ and torsion $\beta_{2}$ for tip apex displacement $\vec{d}\;$via the sensitivity matrix of the angles of HS2.

(5) $$\begin{array}{c} \left(\begin{array}{c} \alpha_{2}\\ \beta_{2} \end{array}\right)\\ =\left[\begin{array}{ccc} 0 & \alpha_{y2}\left(\frac{c_{zy}\sin\left(\varphi \right)+c_{zz}\cos\left(\varphi \right)}{c_{det}}\right)+\alpha_{z2}\left(\frac{c_{zy}\cos\left(\varphi \right)-c_{zz}\sin\left(\varphi \right)}{c_{det}}\right) & \alpha_{y2}\left(\frac{c_{yy}\sin\left(\varphi \right)+c_{yz}\cos\left(\varphi \right)}{c_{det}}\right)+\alpha_{z2}\left(\frac{c_{yy}\cos\left(\varphi \right)-c_{yz}\sin\left(\varphi \right)}{c_{det}}\right)\\ \beta_{x2}/c_{xx} & 0 & 0 \end{array}\right]\left(\begin{array}{c} x\\ \begin{array}{c} y\\ z \end{array} \end{array}\right)\; \end{array}$$

The dependency of HS1 and HS2 regarding inclination angles and displacements is described in detail by the mechanical model in [10]. The model does not consider the small compression or elongation of the 3D-Nanoprobes flexure hinges in the *y*-direction. 

The interferometer measures the displacement $z_{int1}$ which consists of two components. The first component is the lateral *z*-displacement of HS1 from the *y*- and *z*-displacements of the CD-tip apex. The second one is the horizontal *x*-displacement multiplied by the torsion angle of HS1. It results in a small *z*-displacement by $x_{1}\;*\;\beta_{1}$. Hence, the measured displacement for small angles is given by

(6) $$z_{int1}={\;z}_{1}+{\;x}_{1}{\;*\;\beta }_{1}.\;$$

The relevant force-dependent displacements of HS1 for the interferometer are therefore given by

(7) $$\left(\begin{array}{c} x_{1}\\ z_{1} \end{array}\right)=\left[\begin{array}{ccc} c_{xx1} & 0 & 0\\ 0 & c_{zy1}\cos\left(\varphi \right)-c_{zz1}\sin\left(\varphi \right) & c_{zy1}\sin\left(\varphi \right)+c_{zz1}\cos\left(\varphi \right) \end{array}\right]\left(\begin{array}{c} F_{x}\\ F_{y}\\ F_{z} \end{array}\right).$$

The compliance matrix coefficient $c_{xx1}$ considers only in plane bending. The inclination angles of HS1 regarding the tip–sample interaction force can be described from [10] by

(8) $$\left(\begin{array}{c} \alpha_{1}\\ \begin{array}{c} \beta_{1}\\ \gamma_{1} \end{array} \end{array}\right)=\left[\begin{array}{ccc} 0 & \alpha_{y1} & \alpha_{z1}\\ \beta_{x1} & 0 & 0\\ \gamma_{x1} & 0 & 0 \end{array}\right]\;\;\left(\begin{array}{c} F_{x}\\ F_{y}\\ F_{z} \end{array}\right).$$

Because the matrix in the equation above has no inverse and because $\gamma_{1}$ cannot be measured, the components are rearranged individually to replace the tip–sample interaction force $\vec{F}\;$in Equation (7). This leads to the angle-dependent displacement matrix for HS1 which is detailed in Equation (9).

(9) $$\left(\begin{array}{c} x_{1}\\ z_{1} \end{array}\right)=\left[\begin{array}{ccc} c_{xx1}\;*\;\left(\beta_{1}/\beta_{x1}\right) & 0 & 0\\ 0 & (c_{zy1}\cos\left(\varphi \right)-c_{zz1}\sin\left(\varphi \right))\;*\;\alpha_{1y}/\alpha_{y1} & (c_{zy1}\sin\left(\varphi \right)+c_{zz1}\cos\left(\varphi \right))\;*\;\alpha_{1z}/\alpha_{z1} \end{array}\right]$$

As shown in Equations (1) and (8), the inclination angles regarding torsion are proportional to elements of the force dependent angle matrix, because of the identical tip–sample interaction force. Hence, the inclination angle $\beta_{1}$ of HS1 can be replaced by the measurable one $\beta_{2}$ of HS2, and one can write

(10) $$x_{1}={\;c}_{xx1}\;*\;\left(\beta_{1}/\beta_{x1}\right)={\;c}_{xx1}\;*\;\left(\beta_{1}/\beta_{2}{\;*\;\beta }_{2}/\beta_{x1}\right)\;\;=\;c_{xx1}\;*\;\left(\beta_{x1}/\beta_{x2}{\;*\;\beta }_{2}/\beta_{x1}\right)\;={\;c}_{xx1}\;*\;\left(\beta_{2}/\beta_{x2}\right)$$

Equation (7) can be rewritten with (9) and (10) to be

(11) $$z_{int1}=(c_{zy1}\cos\left(\varphi \right)-c_{zz1}\sin\left(\varphi \right)){\;*\;\alpha }_{1y}/\alpha_{y1}+(c_{zy1}\sin\left(\varphi \right)+c_{zz1}\cos\left(\varphi \right)){\;*\;\alpha }_{1z}/\alpha_{z1}+{\;x}_{1}{\;*\;\beta }_{2}{\;*\;\beta }_{x1}/\beta_{x2}$$

The measured displacement of a virtual tip on HS1 for a pure *z*-displacement is defined with Equation (11) as

(12) $$z_{tipz1}=(c_{zy1}\sin\left(\varphi \right)+c_{zz1}\cos\left(\varphi \right)){\;*\;\alpha }_{1z}/\alpha_{z1}$$

Indicated by Figure 4, the measured $z_{int}$ value of the interferometer of HS1 $z_{int1}$ is also influenced by a tip displacement in the *y*-direction. This influence is described by Equations (9) and (11) and is subtracted to receive the *z*-dependent displacements $z_{tipz1}$ of HS1.

(13) $$z_{tipz1}=z_{int1}-(c_{zy1}\cos\left(\varphi \right)-c_{zz1}\sin\left(\varphi \right)){\;*\;\alpha }_{1y}/\alpha_{y1}-x_{1}{\;*\;\beta }_{2}{\;*\;\beta }_{x1}/\beta_{x2}$$

The probe is optimized and calibrated for a $\alpha_{2z}\left(F_{z}\right)=0$. In general, the ratio $\frac{\alpha_{1}}{\alpha_{2}}$ is given by the sum of the *y*- and *z*-force-induced bending angles $\frac{\alpha_{1y}+\alpha_{1z}}{\alpha_{2y}+\alpha_{2z}}\;.\;$In the *y*-direction, the corresponding angles are given by$\;\alpha_{1z}\;=0\;and\;\alpha_{2}=\alpha_{2y},\;$by calibration.

The ratio becomes $\frac{\alpha_{1}}{\alpha_{2}}=\frac{\alpha_{1y}}{\alpha_{2y}}=\frac{\alpha_{y1}}{\alpha_{y2}},$ because the inclination angles have the same ratio as the coefficients of the matrix, which are received from the model. 

This results in the HS1 virtual tip-related displacement

(14) $$z_{tipz1}=z_{int1}-(c_{zy1}\cos\left(\varphi \right)-c_{zz1}\sin\left(\varphi \right)){\;*\;\alpha }_{2}/\alpha_{y2}-x_{1}{\;*\;\beta }_{2}{\;*\;\beta }_{x1}/\beta_{x2}$$

with the calculated compliances of Equation (7) from a virtual tip on HS1 in the *z*-direction $c_{zz1}\;$and the compliance of the 3D-Nanoprobe including the CD tip $c_{zz}$, the CD tip displacement of the 3D-Nanoprobe $z_{tip}$ is given by the ratio

(15) $$\frac{c_{zz1}}{c_{zz}}=\frac{z_{tipz1}}{z_{tip}}$$

Rearranging and implementing Equation (14) into the equation above results in the inclination angle and the measured *z*-displacement of HS1 that is dependent on the CD tip *z*-displacement

(16) $$z_{tip}={\;z}_{int1}\frac{c_{zz}}{c_{zz1}}-(c_{zy1}\cos\left(\varphi \right)-c_{zz1}\sin\left(\varphi \right))\;*\;\frac{\alpha_{2}}{\alpha_{y2\;}\;*\;}\frac{c_{zz}}{c_{zz1}}-{\;c}_{xx1}\;*\;\left(\beta_{2}/\beta_{x2}\right){\;\;*\;\beta }_{2}\frac{\beta_{x1}}{\beta_{x2}}\frac{c_{zz}}{c_{zz1}}$$

This general model has only used modelled properties of the 3D-Nanoprobe so far. Obviously, the detected signals depend on the AFM head used, which is considered in the following. The optical lever signals are simulated by a collimated Gaussian beam of 2.24 mm waist in diameter which passes an aperture of 2.5 mm. The beam is collected by a QPD. For the components used, the normalized signal changes by about $1.3\;*\;10^{-3}$/µm beam displacement for small angles. It is normalized by the summation signal $V_{SUM}$ of the QPD. The measured change in inclination angles of HS2 of the 3D-Nanoprobe is registered as a displacement $s_{OPT}$ on the QPD via the focal length $F_{OPT}$ of 4 mm of the microscope objective. The measured displacement $s_{OPT}$ of the beam for a dual-optical lever for small angles is given by $\Delta \;s_{OPTx}\;=\;2\beta_{2}\;F_{OPT}$ and $\Delta \;s_{OPTy}=\;2\alpha_{2}\;F_{OPT}$. The signal of the QPD circuit $S_{QPDx}\;and\;S_{QPDy}$ can be calculated as

(17) $$S_{QPDx}={\;V}_{SUM}\;*\;10.08{\;*\;\beta }_{2}\;and$$

(18) $$S_{QPDy}={\;V}_{SUM}\;*\;10.08{\;\;*\;\alpha }_{2}$$

The interferometer signals are used to calculate the relative displacement. The built-in field programmable gate array (FPGA) of the interferometer calculates the real-time signal and outputs it via a digital-to-analog converter with $\phi_{int}\;=\;10\;\mathrm{nm}/V$, making this interferometer differential. The expected signal $S_{INTz}$ is given by

(19) $$S_{INTz}=\frac{{\Delta z}_{int1}}{\phi_{int}}$$

Equations (17) and (18) of the simulated signals along with Equation (19) are rearranged and implemented into the CD tip displacements of Equations (5) and (16). This results in the 3D CD tip displacements of the 3D-Nanoprobe.

(20) $$x_{tip}=\frac{S_{QPDx}}{V_{SUM}\;*\;10.08\;*\;\frac{\beta_{x2}}{c_{xx}}}$$

(21) $$y_{tip}=\frac{S_{QPDy}}{V_{SUM}\;*\;10.08\;*\;\left(\alpha_{y2}\left(\frac{c_{zy}\sin\left(\varphi \right)+c_{zz}\cos\left(\varphi \right)}{c_{det}}\right)+\alpha_{z2}\left(\frac{c_{zy}\cos\left(\varphi \right)-c_{zz}\sin\left(\varphi \right)}{c_{det}}\right)\right)}$$

(22) $$\begin{array}{c} z_{tip}=\phi_{int}{\;*\;S}_{INTz}\;\frac{c_{zz}}{c_{zz1}}-(c_{zy1}\cos\left(\varphi \right)-c_{zz1}\sin\left(\varphi \right))\;*\;\frac{S_{QPDy}}{\alpha_{y2\;}{\;*\;V}_{SUM}\;*\;10.08}\frac{c_{zz}}{c_{zz1}}-\left(c_{xx1}\;*\;\frac{S_{QPDx}}{\beta_{x2}{\;V}_{SUM}\;*\;10.08}\;\pm \;x_{offset}\right)\;*\\ \frac{S_{QPDx}}{V_{SUM}\;*\;10.08}\frac{\beta_{x1}}{\beta_{x2}}\frac{c_{zz}}{c_{zz1}}\; \end{array}$$

The $x_{offset}$ is the lateral offset position of the interferometer spot on HS1 in the *x*-direction, which might appear in practice. Rewriting Equations (20)–(22) into a calibration matrix form results in

(23) $$\begin{array}{c} (\begin{array}{c} x_{tip}\\ y_{tip}\\ z_{tip} \end{array})\;=\left[\begin{array}{ccc} \frac{1}{V_{SUM}\;*\;10.08\;*\;\frac{\beta_{x2}}{c_{xx}}}\; & 0 & 0\\ 0 & \frac{1}{V_{SUM}\;*\;10.08\;*\;\left(\alpha_{y2}\left(\frac{c_{zy}\sin\left(\varphi \right)+c_{zz}\cos\left(\varphi \right)}{c_{det}}\right)+\alpha_{z2}\left(\frac{c_{zy}\cos\left(\varphi \right)-c_{zz}\sin\left(\varphi \right)}{c_{det}}\right)\right)} & 0\\ -\frac{\pm x_{offset}}{V_{SUM}\;*\;10.08}\frac{\beta_{x1}}{\beta_{x2}}\frac{c_{zz}}{c_{zz1}} & -\frac{\left(c_{zy1}\cos\left(\varphi \right)-c_{zz1}\text{sin}\left(\varphi \right)\right)}{\alpha_{y2\;}\;*\;V_{SUM}\;*\;10.08}\frac{c_{zz}}{c_{zz1}} & \phi_{int}\;\frac{c_{zz}}{c_{zz1}} \end{array}\right](\begin{array}{c} S_{QPDx}{}^{2}\\ S_{QPDy}{}^{2}\\ S_{INTz}{}^{2} \end{array})+\\ \left[\begin{array}{ccc} 0 & 0 & 0\\ 0 & 0 & 0\\ -\frac{c_{xx1}}{\beta_{x2}\;V_{SUM}{}^{2}10.08^{2}}\frac{\beta_{x1}}{\beta_{x2}}\frac{c_{zz}}{c_{zz1}} & 0 & 0 \end{array}\right]\left(\begin{array}{c} S_{QPDx}{}^{2}\\ S_{QPDy}{}^{2}\\ S_{INTz}{}^{2}\; \end{array}\right) \end{array}$$

The higher order term of the torsion angle has a very small influence on the interferometer and can be neglected. This results in the sensitivity matrix for calibration $S_{cal}$ of the 3D-Nanoprobe in the coordinate system of the probe

(24) $$\left(\begin{array}{c} x_{tip}\\ \begin{array}{c} y_{tip}\\ z_{tip} \end{array} \end{array}\right)=S_{cal}\left(\begin{array}{c} S_{QPDx}\\ S_{QPDy}\\ S_{INTz} \end{array}\right).$$

Machine coordinates are received by transforming the calibrated 3D-Nanoprobes coordinate system into the stage/machine coordinate system $\left(\begin{array}{c} x_{stage}\\ y_{stage}\\ z_{stage} \end{array}\right)$, by rotating backwards around the *x*-axis by the determined $\varphi_{tilt}$ which is, in the ideal case, the incident angle φ.

(25) $$\left(\begin{array}{c} x_{stage}\\ y_{stage}\\ z_{stage} \end{array}\right)=\left[\begin{array}{ccc} x_{tip} & 0 & 0\\ 0 & y_{tip}\cos\left(-\varphi_{tilt}\right) & -z_{tip}\sin\left(\varphi_{tilt}\right)\\ 0 & -y_{tip}\sin\left(\varphi_{tilt}\right) & z_{tip}\cos\left(-\varphi_{tilt}\right) \end{array}\right]$$

The angle $\varphi_{tilt}$ is defined during calibration by the orientation of the probing vector $\vec{d}$ with $\alpha_{2}\left(\vec{d}\right){}^{!};=$ 0.

A typical 3D-Nanoprobe based on a CDR120 CD-tip cantilever and a beam location offset of the interferometer beam on HS1 of $x_{offset}=\pm 3\;µm\;$and a signal in mV has typically has the following model-based calibration coefficients for a tip displacement in nm of

(26) $$\begin{array}{c} \left(\begin{array}{c} x_{tip}\\ \begin{array}{c} y_{tip}\\ z_{tip} \end{array} \end{array}\right)\;\;=\;\left[\begin{array}{ccc} 0.277\;\pm \;0.024 & 0.00 & 0.00\\ 0.00 & 0.213\;\pm \;0.015 & 0.00\\ 0.001\pm \;0.001 & -0.082\pm \;0.017 & 0.021\;\pm \;0.001 \end{array}\right]\left(\begin{array}{c} S_{QPDx}/V_{SUM}\\ S_{QPDy}/V_{SUM}\\ S_{INTz}\; \end{array}\right)\\ +\left[\begin{array}{ccc} 0.00 & 0.00 & 0.00\\ 0.00 & 0.00 & 0.00\\ \;0.00006\;\pm \;0.00003 & 0.00 & 0.00 \end{array}\right]\left(\begin{array}{c} S_{QPDx}{}^{2}/V_{SUM}{}^{2}\\ S_{QPDy}{}^{2}/V_{SUM}{}^{2}\\ S_{INTz}{}^{2}\; \end{array}\right) \end{array}$$

As shown by (25), higher order calibration coefficients resulting from the torsion angle of HS1 can be neglected.

The calibrated 3D-Nanoprobe used for measurements in Section 3 is taken as an example and shows linear calibration coefficients of

$$S_{cal\_exp}=\;\left[\begin{array}{ccc} 0.4150 & -0.0190 & 0.00\\ -0.0050 & 0.3230 & -0.0013\\ -0.0090 & -0.1150 & 0.0175\; \end{array}\right]$$

The experimental calibration results of the 3D-Nanoprobe indicate that the model can be used to determine the sensitivity of the 3D-AFM head and the 3D-Nanoprobe. The offset of the horizontal coefficients (*x*- and *y*-direction) might come from a slightly softer CD tip than expected, whose stiffness cannot be determined separately at the moment. There are also influences of the compression and elongation of the compliant structures and manufacturing uncertainties in the FIB process. The *z*-calibration coefficient of the interferometer is influenced by its position on HS1. The closer the spot is located to the tip region, the larger the signal and the smaller the calibration coefficient. The model uses the center position of HS1. To align the interferometer spots on the 3D-Nanoprobe the visible so-called probe beam (wavelength about 650 nm) is used. This beam is part of the interferometer device (from the SmarAct Picoscale company) and offers a rough estimation of the actual beam (1550 nm center wavelength) location of the interferometer spot. The beam spots can be offset by a few µm to each other, which can lead to an estimated deviation of about 20%, of experimental interferometer coefficient 0.021 ± 0.001 to the expected 0.0175. Other differences and crosstalk come from the lateral offset of the tip’s center position and from the slight rotation of the mounted 3D-Nanoprobe. The 3D-Nanoprobe chip has no alignment structure. Consequently, imperfect mounting of a few degrees occurs, mainly around the vertical *z*-axis of the probe. However, the crosstalk is calibrated and therefore is not an issue.

#### 2.2.2. Calibration of the 3D-Nanoprobe

**Figure 5:**
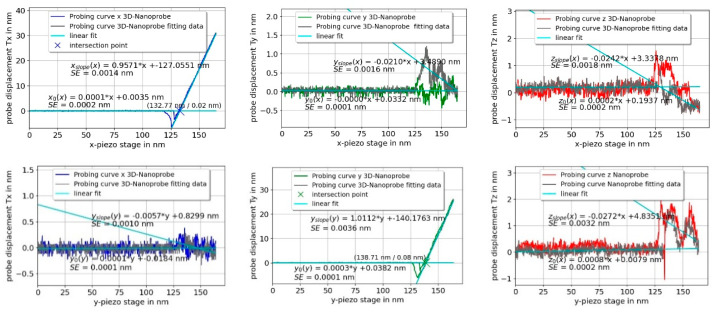

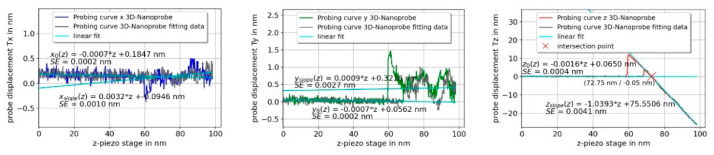
Full set of calibration curves for a 3D-Nanoprobe in the orthogonal *x*-, *y*-, and *z*-directions. Each curve shows the calibrated displacement of the probe over the displacement of the stage. Each curve is fitted by linear fits and the slopes are taken to determine the quality of the calibration. The probing curves are full interaction curves with retraction (snap-in and pull-off clearly visible, for instance, for *z*-probe displacement while probing in the *z*-direction, bottom right).

The calibration procedure of the 3D-Nanoprobe starts with the *z*-direction. The orientation of the probing vector is adjusted in the *zy*-plane until $\alpha_{2}\left(F_{z}\right)\;=0$, while probing on a horizontal plane surface. This denotes the *z*-axis of the 3D-Nanoprobe. The angle between the orientation of the stage *z*-axis and the *z*-axis of the 3D-Nanoprobe is the angle $\varphi_{tilt}$, under ideal circumstances, it is the incident angle (8°), as mentioned previously. Full 3D selectivity is reached, by probing in the three spatial orthogonal directions of the 3D-Nanoprobe and adjusting the calibration factors in the matrix $S_{cal}$. The slope of the probing curve in the probing direction is adjusted to 1 and the corresponding slopes of the two orthogonal directions are adjusted to zero. The calibration shown in Figure 5 was done manually $(\varphi_{tilt\;}$= 8.5°) on the rigid part of IVPS100-PTB samples [22]. 

Figure 5 shows one set of calibration probing curves for probing in the vector approach probing (VAP) mode [8,9] in the *x*-, *y*-, and *z*-direction perpendicular to a plane surface. The unbiased, non-displaced CD tip positions of the 3D-Nanoprobe are fitted by the $x_{0},$ $y_{0}$, and $z_{0}$ functions, shown in Figure 5. The linear slopes of the probing curves $x_{slope},$ $y_{slope}$, and $z_{slope}$ denote the dependency of a measured displacement of the 3D-Nanoprobe’s CD tip to the displacement of the stage while being in contact with the sample. The intersection point of the linear fit of the unbiased tip position with the linear fit of the linear fitted slope marks the probed surface point of the sample.

The slopes are compared to determine the quality of the calibration factors of the calibration process. Periodic non-linearities of the interferometer, mainly caused by the microscope objective of the detection unit (described in Section 4), can be addressed as one of the main uncertainties of the slopes. Nevertheless, one criterion of the 3D-Nanoprobe is the selectivity for the calibration directions. It is determined by normalizing the slopes of each *x*-, *y*-, and *z*-direction. An average of five probing curves is taken, which results in a selectivity matrix $S_{sel}$ for a 3D-Nanoprobe with a selectivity ratio of about 50:1 for manual calibration.

$$\left(\begin{array}{c} \Delta x_{stage}\\ \begin{array}{c} \Delta y_{stage}\\ \Delta z_{stage} \end{array} \end{array}\right)=\left[\begin{array}{ccc} \;1.000\;\pm \;0.004\; & -0.016\;\pm \;0.005 & 0.001\;\pm \;0.004\\ 0.003\;\pm \;0.004 & 1.000\;\pm \;0.001 & -0.001\;\pm \;0.002\\ -0.010\;\pm \;0.006 & 0.019\;\pm \;0.003 & 1.000\;\pm \;0.005 \end{array}\right]\;\left(\begin{array}{c} \Delta x_{tip}\\ \begin{array}{c} \Delta y_{tip}\\ \Delta z_{tip} \end{array} \end{array}\right)\;$$

The slopes of the displacements in probing the directions *x*, *y*, and *z* are 1.010 ± 0.004, 1.032 ± 0.001 and −1.039 ± 0.005 respectively, indicating a successful calibration. As has been demonstrated, the 3D-Nanoprobe has full 3D sensitivity.

### 2.3. The 3D-AFM Head

**Figure 6:**
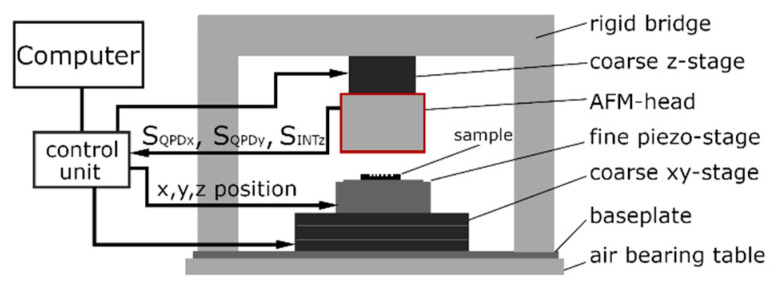
Sample scanning AFM setup used for testing the new 3D-AFM head.

The schematic layout of the sample-scanning AFM setup built by PTB staff is shown in Figure 6. It is used for the calibration of the 3D-Nanoprobe and measurements. The setup consists of a fixed bridge frame, which is mounted on the baseplate, stages for positioning, and a 3D-AFM head that was also built by PTB staff. The mechanical coarse stage for *xy*-positioning is mounted on the baseplate and carries the six degrees of freedom (DOFs) positioning piezo stage with the sample. The so-called 3D-AFM head (designated as AFM head in Figure 6) is mounted on the coarse *z*-stage, which is suspended from the fixed bridge. A control unit (e.g., a digital signal processor (DSP)) receives the signals $S_{QPDx}$, $S_{QPDy}$, $S_{INTz}$, and $V_{SUM}$ from the 3D-AFM head, controls the probing in 3D, (e.g. tip–sample distance) and is connected to a computer with a user interface.

**Figure 7:**
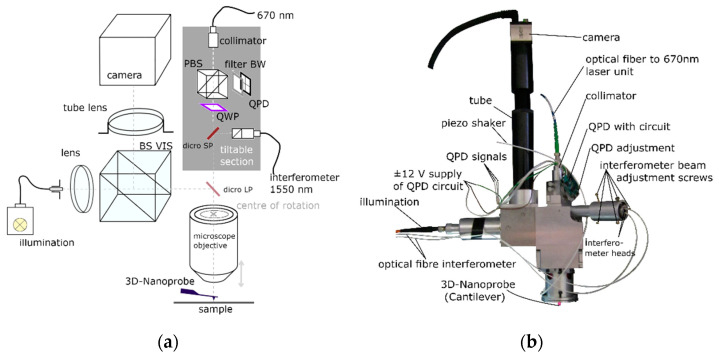
Schematic drawing of the true 3D-AFM-head (**a**) consisting of an optical microscope and a detection unit. Both share the same microscope objective. Image of the 3D-AFM-head (**b**).

The electronics, detectors, data acquisition, and regulation (control unit) are also built by PTB staff as well as being based on earlier developments [8] and are not explained in detail in this paper. The focus is on the self-build 3D-AFM head, marked red in Figure 6 and detailed in Figure 7. It is designed to detect the three DOFs of the 3D-Nanoprobe’s tip apex and consists of an optical microscope built by PTB staff and an optical detection unit (grey) in Figure 7a. The optical microscope is used for sample inspection and to focus and locate the laser spots of the detection unit on the 3D-Nanoprobe with the camera image. The optical path of the optical microscope and 3D-Nanoprobe detection unit use the same microscope objective (Mitutoyo 50× Plan Apo NIR) in parallel and are separated by a dichroic long pass filter (dicro LP).

The 3D-Nanoprobe detection unit in Figure 7a consists of two parts, the built is shown in Figure 7b. One part of the detection unit is a dual-optical lever to register bending and torsion angles of an in-focus mounted probe (cantilever or 3D-Nanoprobe). A laser beam is collimated from a self-built superluminescent light emitting diode (SLED) unit with a center wavelength of about 670 nm. It passes a polarizing beam splitter (*PBS*) and is circular polarized by a quarter wave plate (QWP) rotated at 45°. The beam then passes a dichroic short pass filter (dicro SP) and a dichroic long pass filter (dicro LP), is coupled into the microscope objective, and is focused and aligned on the reflective-coated reverse side of the probe. The reflected laser beam from the probe’s reverse side is collimated by the microscope objective. It then passes the filter dicro LP and dicro SP filters and is linear polarized by the QWP. The linear polarized beam is coupled out by the *PBS* onto a four-quadrant photodiode detector (QPD). During testing, the SLED beam with 670 nm wavelength used for the optical lever showed a change in polarization passing the dicro SP filter, acting like the built-in QWP. Because of the dicro SP filter properties, the QWP was removed from the setup but has been left in the drawing for more clarity. 

**Figure 8:**
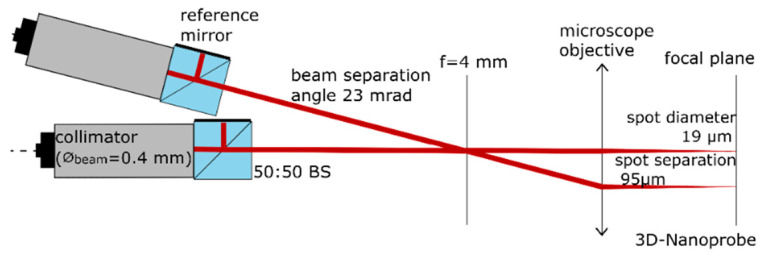
Sketch of the interferometer unit with two interferometer heads (C01, the SmarAct Picoscale company, Oldenburg, Germany). The measured displacements are subtracted in real time, making this unit a differential interferometer.

The second part of the detection unit is the interferometer unit to detect the vertical *z*-displacement. It is shown in detail in Figure 8. Two C01 50:50 (SmarAct Picoscale) interferometer heads are used to measure the probes displacement. One interferometer head is used to measure the displacement of the tip region and the second head is aimed at the base of the probe to measure the displacement of the chip of the probe (bulk), as indicated in Figure 3 above. The difference between these measured displacements is calculated in real time by an internal FPGA of the SmarAct Picoscale’s device, resulting in the measured displacement of the probe’s tip region. This greatly reduces the influence of the environmental conditions, such as temperature drifts, ambient air pressure changes, and fluctuations in humidity and CO_2_ levels in the atmosphere, to about 10%, because the optical paths (dead paths) are almost identical.

The beam separation of about 95 µm is determined by the distance from the tip region to the base region of the 3D-Nanoprobe. This is achieved by coupling the two interferometer heads into the 3D-AFM head with a beam separation angle of about 1.36° (23 mrad) to each other. The interferometer heads are mounted in an *Invar* sleeve, which reduced the influence of thermal drift by a factor of about 20 compared to a Duralumin sleeve. The rest of the setup is made from Duralumin. Limited space and distance restrictions lead to a geometr*y*-based offset in the length of optical paths of about 14 mm. 

## 3. Results and Discussion

The 3D-Nanoprobe was tested for manufactured stiffness and probing repeatability. It was also applied for measurements of CD values using the VAP method [8,9]. In addition, pitch and roughness measurements were performed.

### 3.1. 3D-Nanoprobe Stiffness Calibration

To calibrate the overall stiffness of the 3D-Nanoprobe, a reference spring based on an micro electro mechanical system (MEMS) device has been used [23]. The calibrated stiffness value of the reference spring $k_{ref}\;=2.571\left(2\right)\;$N/m has been compared with the stiffness of the 3D-Nanoprobe via the slope m of two probing curves. The first curve is carried out on a rigid bulk part of the support chip of the reference spring; the second probing is done on the compliant reference spring. The stiffness of the probe $k_{probe\_i}$ is calculated by Equation (27).

(27) $$k_{probe\_i}=\left(\frac{m_{rigid\_i}\;}{m_{spring\_i}}-1\right){\;k}_{ref},\;i\;=\;x,\;y,\;z.$$

**Figure 9:**
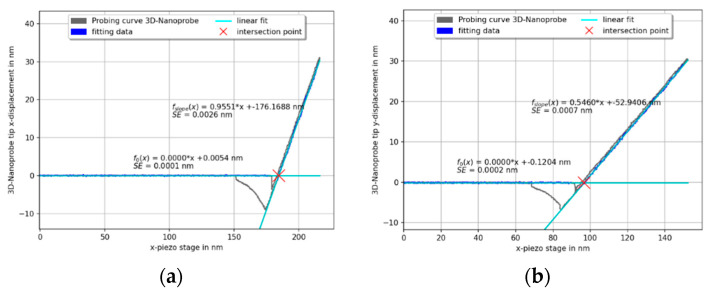
Two full probing curves (approaching and receding) from the bulk of the rigid support chip (**a**) and from the spring (**b**) of the reference spring; SE is the standard error of the slope.

Two probing curves with fitted slopes $m_{rigid}=\;0.9551\pm \;0.0052$ and $m_{spring}=\;0.5460\pm \;0.0004\;$are shown in Figure 9a,b. For each slope $m_{rigid\_i}$ and$\;m_{spring\_i}$, an average of five repeated measurements (probing curves) were taken in each spatial direction of the 3D-Nanoprobe.

In total, three 3D-Nanoprobes were taken to determine the manufactured stiffness, and these were compared with the finite element method (FEM) simulated stiffness. The results are shown in Table 1. 3D-Nanoprobe A shows an offset in the finite element method (FEM) designed stiffness to the manufactured stiffness of about −0.41 N/m, −0.47 N/m, and −0.81 N/m for *x*, *y*, *z* respectively. The stiffnesses are determined by $k_{x}\;=\;1.722\;\pm \;0.083$ N/m, $k_{y}\;=\;1.511\;\pm \;0.034\;N/m\;and\;k_{z}\;=\;1.64\;\pm \;0.16$ N/m. Therefore 3D-Nanoprobe A shows a very good isotropic ratio of the stiffnesses of 1.05:0.91:1.00 for the *x*-, *y*-, and *z*-direction, respectively. 3D-Nanoprobe B shows similar values and only has a small offset of the measured stiffness to the FEM modelled stiffness of about 0.09 N/m, 0.19 N/m, and −0.41 N/m for the *x*, *y*, and *z*, respectively, from the desired stiffness value. The stiffnesses are determined by $k_{x}\;=\;2.016\;\pm \;0.024$ N/m, $k_{y}\;=\;2.036\;\pm \;0.070\;N/m$, and $k_{z}\;=\;2.311\;\pm \;0.086$ N/m, thus the ratio of the stiffness is 0.87:0.88:1.00 for the *x*-, *y*-, and *z*-direction, respectively. 3D-Nanoprobe C has some deviation from the optimized design values. It has an offset of about 0.50 N/m, 0.04 N/m, and −1.12 N/m for *x*, *y*, and *z*, respectively, regarding the desired FEM modeled stiffness value. The stiffnesses are determined by $k_{x}\;=\;2.719\;\pm \;0.067$ N/m, $k_{y}\;=\;2.461\;\pm \;0.065\;N/m$, and $k_{z}\;=\;1.198\;\pm \;0.099$ N/m. Accordingly, the ratio of the stiffness is 2.27:2.05:1.00 for the *x*-, *y*-, and *z*-direction, respectively. 3D-Nanoprobe C shows an offset of about 50% for stiffness $k_{z}\;$, which can be explained by FH1 manufactured in too thin a manner (about 100 nm). The offset in stiffness of the *x*- and *y*-direction compared to the design values is indicated by FH2 being manufactured in a way that was slightly too thick (a few ten nanometers). This assumption of an unbalanced geometry ratio of HS1 to HS2 is supported by a smaller determined $\varphi_{tiltC}\;\mathrm{of}\;6.8^{{}^{\circ}}$, which was determined during previous a calibration. Nanoprobes A and B show almost identical values of the $\varphi_{tiltA}=\;8.3^{{}^{\circ}}$ and $\varphi_{tiltB}=\;8.5^{{}^{\circ}}$ compared with the incident angle of the 3D-Nanoprobe used in this the setup, which means that the manufactured geometry of FH1 to FH2 is close to the optimized modeled geometry.

Deviations in the range of about 25% of the modelled stiffness values to the measured stiffness values can be addressed mainly by two reasons. The first of these is due to deviations of FH1 and FH2 in geometry caused by the manufacturing process, because of the small dimension of the flexure hinges. Those dimensions have a strong influence on the overall stiffness. The second reason is an unknown exact CD-tip geometry which is individual for each 3D-Nanoprobe and may also change during measurement by tip wear. In the FEM model, a fixed geometry with nominal values has been used. The deviation of the FEM-based modeled stiffness values that do not reach the isotropic ratio of stiffness result from deviations to the model and a small tip overhang of the used structured CD cantilevers used. This tip overhang is a strong boundary and affects the optimization process. Overall, this indicates the importance of an optimization process for each 3D-Nanoprobe as well as and gathering experience in FIB-manufacturing process. During stiffness calibration, it was found that the manufactured 3D-Nanoprobe is very robust and flexible owing to its compliant flexure hinge structure.

### 3.2. Probing Repeatability

**Figure 10:**
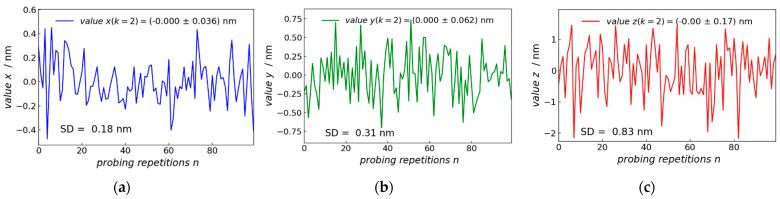
Measurement repeatability: 100 times probing on the same spot on the rigid bulk material of an IVPS100-PTB structure in (**a**) *x*-, (**b**) *y*-, and (**c**) *z*-direction, *x*- and *y*-coordinate are probed on the sidewall (SD = standard deviation).

To investigate the probing repeatability of the 3D-Nanoprobe in orthogonal *x*-, *y*-, and *z*-directions, three orthogonal plane surfaces are probed separately. The probing is performed on two IVPS100-PTB type samples [22]. IVPS100-PTB (chip dimension 6 × 6 mm) contained four quadrants with 25 structured and numbered cells and was made of silicon crystal. Each cell contained five parallel lines named as S1, S2, S3, S4, and S5 with a nominal width of about 50, 70, 90, 110, and 130 nm with vertical sidewalls and a pitch of about 500 nm. The sample IVPS100-PTB no. 0301, which is called IVPS0301 in the following, was aligned with the coordinate system of the piezo stage (the coordinate system of the machine) with the lines aligning along the *x*-axis. The second sample IVPS100-PTB no. 0205 called IVPS0205 in the following, was mounted and rotated by 90 degrees around the vertical *z*-axis to the IVPS0301. The IVPS0205 aligned with the *y*-axis. To avoid any bias of the lateral stiffness of the IVPS100-PTB lines in the *x*- and *y*-direction, rigid parts of the sidewalls were probed. The 3D-probing capability of the 3D-AFM head was tested with a 3D-Nanoprobe based on CD tip cantilever CDR120 (manufactured by the Nanosensors company) with a probing speed of 1000 nm/s. Figure 10a–c show the probing in the *x*-, *y*-, and *z*-direction, respectively for 100 points. The standard deviations in the *x*-direction is 0.18 nm, while the *y*- and *z*-directions are kept constant, and the standard deviation remains below 0.025 nm and 0.010 nm in the *y*- and *z*-direction, respectively. The measurement in the *y*-direction shows a standard deviation of 0.31 nm, while the standard deviation in the constant *x*- and *z*-direction (not shown) are below 0.015 nm and 0.009 nm, respectively. Probing in the *z*-direction shows a larger standard deviation of 0.83 nm, while the standard deviation of the constant *x*- and *y*-direction (not shown) remains below 0.033 nm and 0.023 nm, respectively. The *y*-direction shows higher values for standard deviation compared to the *x*-direction, because it is influenced by the interferometer signal as shown in the matrix of Equation (25). The interferometer mainly measures the *z*-direction of the 3D-Nanoprobe CD tip displacement and has the largest standard deviation because of the interferometer noise. The performance of the interferometer is compromised due to several optical interfaces in the microscope objective, resulting in stray light which causes nonlinearities. Moreover, the interferometer is influenced by ambient environmental conditions. It should be noted that all these measurements are also influenced by the position noise of the piezo stage.

### 3.3. Repeatability Measurements

**Figure 11:**
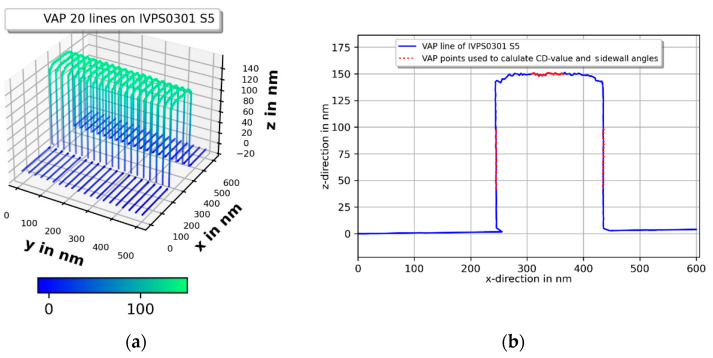

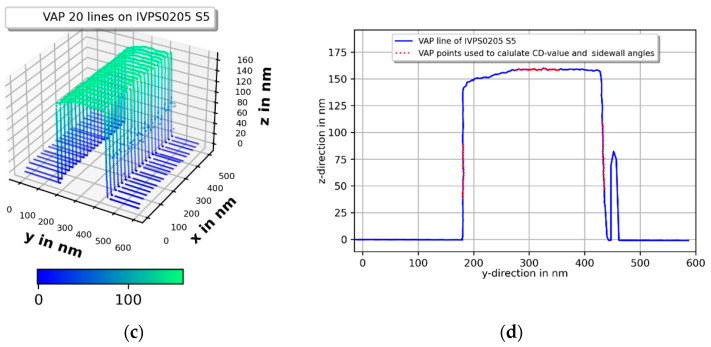
Vector approach probing (VAP) measurements (raw data) of the two IVPS100-PTB structures IVPS0301 in (**a**) and IVPS0205 in (**c**) orthogonally mounted to each other. One measured line is shown in (**b**,**d**) for the *x*- and *y*-direction, respectively.

Repeatability is investigated by measuring the CD values of the widest line structure S5 of the two IVPS100-PTB type samples IVPS0301 and IVPS0205. IVPS0301 has been mounted to measure the CD value in the *x*-direction. IVPS0205 has been mounted to measure the CD value in the *y*-direction. An area of 600 nm (fast scan) × 500 nm (slow scan) was scanned with 20 lines per measurement in the *x*- and *y*-direction 12 times. Each individual measurement included the search of the individual S5 structure with a so-called vector-probing (VP)-line scan, which is described in detail in [8]. This VP-line scan scans the sample by probing only in *z*-direction, similar to the so-called DT mode or the step-in mode in [24,25], without dithering of the probe. Based on the VP-line scan points the probing vectors are calculated for the so-called Mustercurve, for the VAP 3D scan. The Mustercurve for the following CD measurements contains a total of 240 points per line: 30 points on the bottom left side on the so called cut-off plane; five points in the bottom left corner, 50 points on the left sidewall; 10 points on the left upper corner, 50 points on the top of the structure; 10 points on the right upper corner; 50 points on the right sidewall; 5 points in the bottom right corner; and 30 points on the bottom right side on the cut-off plane. The probing speed was set at 1000 nm/s. Because the IVPS sample is structured deeper (about 900 nm) than the CD-tip lengths (about 600 nm) a so-called cut-off plane is applied, to protect the tip and sample. It is a virtual plane to limit the probing region in the *z*-direction and allow only valid points above this virtual plane. To calculate the middle CD value, 20 points (marked in Figure 11) of the middle region of the left and right sidewall are used. Hence, corner rounding, and higher order tip effects are reduced. The CD value is calculated using the points from no. 50 to point no. 70 the left sidewall and then using point no. 170 to point no. 190 on the right sidewall as shown in Figure 11. Two linear fits are performed with these points for each side, and the values of these fits at a depth of 75 nm are used as the middle CD-value. The region of point no. 110 to point no. 130 is used for a linear fit to receive the *z*-value as a reference per line to guarantee a consistent *z*-value depth. For the *y*-direction, the top points are shifted by five points from no. 110 to no. 115 and from no. 130 to no. 135 to avoids tip-related effects. Because there is always a small slope of the linear fits and unequal sidewall angles the CD value is determined iteratively. All measurements are shown without tip correction. Figure 11a,c shows one raw data set for each of the two IVPS100-PTB samples in 3D. Figure 11b,d shows the 2D projection of one measured line for the *x*- and *y*-direction, without any correction. The upper part of the S5 structure is measured and the cut-off plane is set at about 150 nm below this upper part. The 2D projection of the IVPS0301 structure shows nearly vertical sidewalls and a drift of about 12 nm for 12 repetitions, mainly in the *z*-direction.

**Figure 12:**
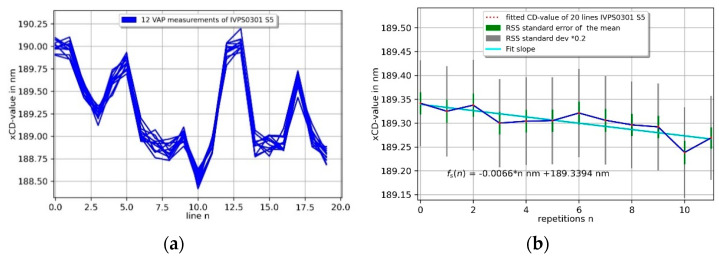

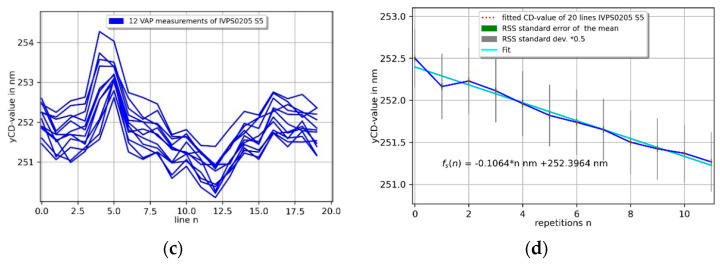
Repetition measurements of two orthogonally mounted IVPS100-PTB structures (IVPS0301 and IVPS0205). A total of 20 lines per measurement were taken and the CD-value is shown for each line on the left side by (**a**,**c**) and for each measurement by (**b**,**d**) on the right side, respectively.

The upper corners of the measured contour in the *x*-direction of Figure 11 show a dip (arrows) in the corner rounding, which might be a snap-in effect by the soft 3D-Nanoprobe during the measurement, or the Mustercurve might have been calculated slightly too narrowly, or the corner radius of the IVPS0301 might have been assumed to be too small. This will be investigated in the future. It might also be a CD tip-shape-related effect, because the probing direction changes at the corner, and consequently, it might not be orthogonal to the surface. The 2D projection of the IVPS0205 in the *y*-direction shows a small drift and CD tip related probing artefacts. The upper left corner shows a sloped plateau from the tip–sample convolution of the blunt CD-tip apex probing the upper left sharp corner of the IVPS0205 structure. The value of the slope is the incident angle $\;\varphi_{Setup}$ of about 8° of the 3D-Nanoprobe. This incident angle also influences the probing of the right sidewall. The CD tip apex has only a small overhang and does not reach the right sidewall at depth, resulting in a measured non-vertical sidewall. The spikes near the lower right corner of the right cut-off plane are from probing in the *z*-direction in the cut-off plane region, while the CD tip’s shaft comes into contact with the upper right corner of the IVPS0205 structure. The spike artefact has no influence on our measurements. These artefacts can be removed in future by using a pretilted CD tip, which compensates for the incident angle. Figure 12 shows the CD values determined for each of the 20 lines per measurement. The xCD value shows very good repeatability in a range of about 0.25 nm, while following the contour of the IVPS0301 sample.

The average xCD value per measurement is shown on the right upper side (b) of Figure 12 and is determined via a line-level fit to be consistent with the CD value per line, the average decrease in CD value per measurement is about −6.6 pm, which results in about −0.33 pm per line. The yCD value shows good repeatablity in following the contour of the IVPS sample in a range of about 1.3 nm. The average yCD-value per measurement is shown in Figure 12 on the lower right side (d) and decreases by about −0.11 nm per measurement which results in about −5.4 pm per line. The main issue is a decreasing CD value in the *y*-direction owing to the fact that the 3D-Nanoprobe has an incident angle of about 8° but has only a small overhang (flared at the tip apex). This has an influence on the probing conditions on the right sidewall in the *y*-direction, because the CD tip’s shaft is used for probing the sharp upper right corner of the IVPS structure. The overall decrease of the average CD values in Figure 12b,d can be explained by tip wear and abrasion processes. Those have an influence on the CD-tip width, and thus the apparent CD value.

**Figure 13:**
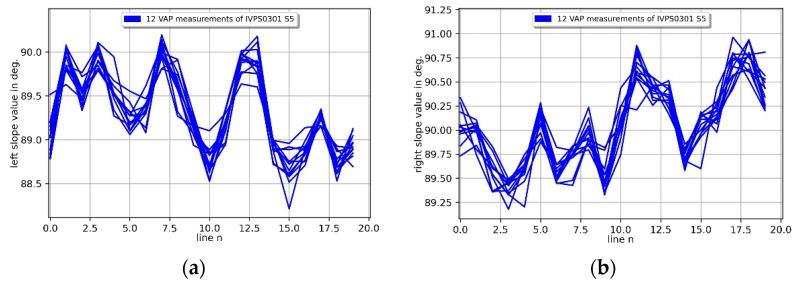

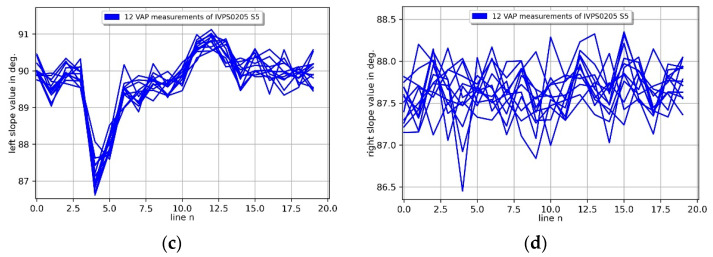
Repetition side wall angle measurements of two orthogonal mounted IVPS100-PTB structures (IVPS0301 (**a**,**c**) and IVPS0205 (**b**,**d**)). The left side wall angles are shown in (**a**,**c**) and the right side wall angles in (**b**,**d**) over 20 lines.

The corresponding sidewall angles are shown in Figure 13. The expected sidewall angle of an IVPS is 90°. The sidewall angle of IVPS0301 in the *x*-direction shows very good agreement by about 89.5° and about 90.0° for the left and right sidewalls in Figure 13a and Figure 13b, respectively. The left sidewall of IVPS0205 in the *y*-direction in Figure 13c shows good agreement with the nominal value of 90° by about 89.8° with good repeatability. The right sidewall of the IVPS0205 in Figure 13d is 87.6° and shows an almost constant value because the CD tip shaft is measured by the sharp corner of the IVPS and not the sidewall of the structure as expected from the previous explanation.

### 3.4. Stability Measurements

**Figure 14:**
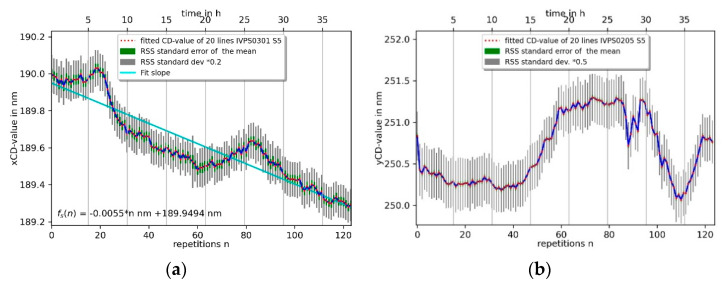
Stability of CD value measurement of two orthogonal mounted IVPS100-PTB structures (IVPS0301 in (**a**) and IVPS0205 in (**b**)) with the 3D-Nanoprobe.

The stability measurements were performed by repeating the same pattern of 20 lines (240 points per line) used for the repeatablity measurement on the same structure, with the same Mustercurve (without VP-line scan), 125 times on the IVPS0301 and IVPS0205. The CD values of the *x*- and *y*-direction of the IVPS0301 and IVPS0205 structure are shown in Figure 14a and Figure 14b, respectively. The measurement took about 40 h in total. A single measurement took 18 min and 41 s, resulting in about 5 points/s. The xCD-value of IVPS0301 has a trend of about −5.5 pm per measurement or −0.29 pm per line, which means a small total decrease of 0.7 nm in CD value. The yCD value of the line of IVPS0205 contains stronger fluctuations and shows two distinct peaks which cannot be attributed to a day and night cycle. A trend line fitted for the sake of completeness would have a slope of 4.6 pm per repetition of a single measurement, but does not represent the curve. The better indicator here is the minimum-to-maximum peak value of about 1.2 nm during the stability measurement.

Both stability measurements show smaller absolute slopes of the trend line regarding the CD values than the repetition measurements, indicating that the VP-line scan to determine the Mustercurve has an influence on the CD value by wearing down the CD tip on the sidewall of the sample [26]. This results in a smaller CD tip width of the 3D-Nanoprobe, and thus in a smaller measured CD value. This can be reduced by using a wear-resistant diamond-like carbon (DLC) coated CD tip.

### 3.5. Pitch and Roughness Measurements

**Figure 15:**
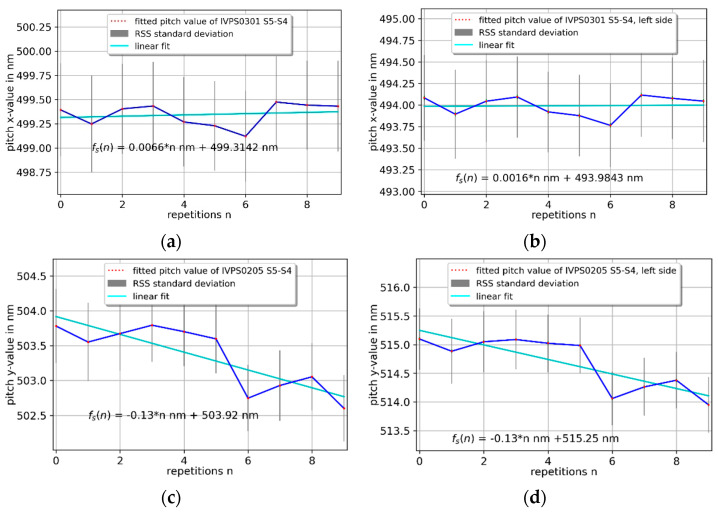
Pitch measurement of the left sidewall with the 3D-Nanoprobe of two orthogonal mounted IVPS100-PTB structures (IVPS0301 (**a**,**b**) and IVPS0205 (**c**,**d**)), showing hinds of asymmetric tip wear in *y*-direction by comparing (**c**,**d**).

**Figure 16:**
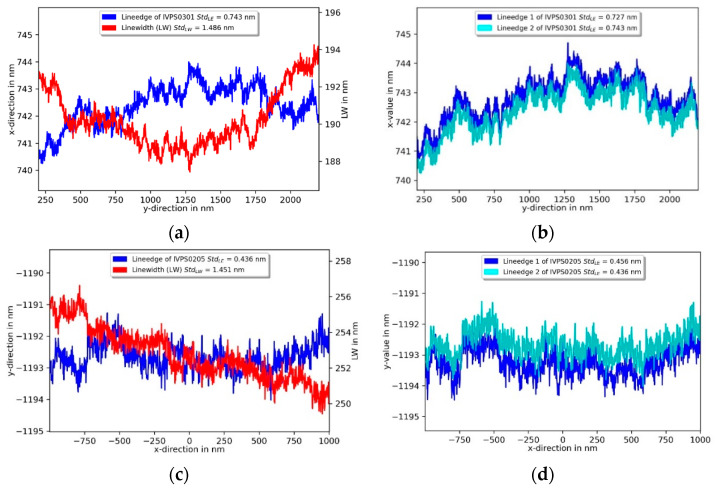
Line edge roughness (LER) and line width (LW) roughness measurement of two orthogonally mounted IVPS100-PTB structures (IVPS0301 and IVPS0205, M35_S5) at a 100 nm depth with the 3D-Nanoprobe in (**a**,**c**). In (**b**,**d**) the lineedge of two repeated measurements are shown.

Pitch measurements were performed by alternating measurements of the widest structure, S5, and the next smaller structure, S4, of both IVPS0301 and IVPS0205. The IVPS100-PTB lines have a nominal pitch of about 500 nm. A VP-line scan with a new Mustercurve has been applied for each measurement repetition. The Mustercurve has the same point pattern (240 points) as for the repetition and stability measurements. The pitch measurement in the *x*-direction in Figure 15a shows very good consistency with a very small increase of 6.6 pm per measurement. The pitch measurement in the *y*-direction in Figure 16c is biased by the tilted CD-tip and shows a bigger change than in the *x*-direction of −127.8 pm per measurement. To evaluate this rather big decrease, this pitch measurement was used to evaluate one-side probing on the left side of the line structures. The distance of the left sidewalls of the line structures S4 and S5 show an increase of 1.6 pm and a decrease of −126.9 pm in Figure 15b,d in the *x*- and *y*-direction, respectively. This indicates a tip abrasion/wear process on one side of the CD tip in *y*-direction possibly during the VP-line scan for each individual alternating measurement. This can be reduced by a wear-resistant DLC CD tip and an improved probing strategy of the VP-line scan.

Besides CD values and pitch measurement, the line edge roughness and linewidth roughness are important quantities to be determined for the quality assurance of the manufacturing process. The roughness parameters were determined on the S5 structure of the IVPS0301 and IVPS0205 in the calibrated 2 µm region by probing along the sidewall for a distance of 2 µm with 500 points/µm at a depth of 100 nm. In the left upper part of Figure 16 the measurement of the IVPS0301 shows the left wall line edge roughness standard deviation S_q_ of 0.75 nm and the corresponding line-width roughness of S_q_ = 1.49 nm. Two repeated measurements of the left line edge in Figure 16b show very good repeatability of the profile in *x*-direction. Figure 16c shows the measurements on the IVPS0205 in the *y*-direction with a left line edge roughness S_q_ of 0.44 nm and the corresponding linewidth roughness standard deviation of 1.46 nm. Two repeated measurements in Figure 16d of the left line edge also show the very good repeatability of the profile in the *y*-direction.

## 4. Summary and Conclusions

In this paper, a focused ion-beam-manufactured cantilever-based probe with a CD tip, the so-called 3D-Nanoprobe, was used for 3D tactile probing. The 3D-Nanoprobe consists of two flexure hinges and two head sections, which are optimized for the isotropic stiffness ratio, increased sensitivity, and 3D-selectivity [10]. Moreover, the mechanical properties of the probe are adapted to the slender and compliant CD tip. In addition, the 3D-Nanoprobe is optimized not to change the orientation (incident angle) of the head section 2 for a displacement or force in the *z*-direction. This allows detection of the spatial displacement directions (*x*,*y*,*z*) of the CD tip apex separately. The 3D-Nanoprobe flared CD tip displacement is detected by a differential interferometer combined with an optical lever.

The manually calibrated 3D-Nanoprobe reached an average selectivity ratio of about 50:1. The sensitivity of the 3D-Nanoprobe, especially for the optical lever, was slightly smaller than expected but this can be explained by a softer CD-tip. The optimized stiffness of the 3D-Nanoprobe was determined by a reference spring. Three manufactured 3D-Nanoprobes (A, B, and C) were tested. Nanoprobe A showed an excellent quasi-isotropic ratio of the stiffnesses of 1.05:0.92:1 for *x*:*y*:*z*, respectively with a stiffness of $k_{z}\;=\;1.64\;\pm \;0.16$ N/m. However, it showed an offset in experimental values to the modelled design values of 0.41 N/m, 0.47 N/m, and 0.81 N/m in *x*, *y*, and *z*, respectively. Nanoprobe B also showed an excellent quasi-isotropic ratio of the stiffness of 0.87:0.88:1 for *x*:*y*:*z*, respectively, with a stiffness of $k_{z}\;=\;2.311\;\pm \;0.089$ N/m. Furthermore, it had only a small offset of 0.09 N/m, 0.19 N/m, and 0.41 N/m compared to the modelled design values in *x*, *y*, and *z*, respectively. Nanoprobe C showed a quasi-isotropic ratio of the stiffness of 2.27:2.05:1 for *x*:*y*:*z*, respectively with a stiffness $k_{z}\;=\;1.198\;\pm \;0.099$ N/m. It showed an offset of −0.50 N/m, 0.04 N/m, and 1.12 N/m of the modeled design values in *x*, *y*, and *z*, respectively. The offset in the *z*-direction is considered as an outlier and can mainly be explained by FH1 which was manufactured sightly too thinly. Despite the variation in the manufacturing and optimization processes all 3D-Nanoprobes were calibrated, and measurements were performed without compromise. During the measurements, it was found that the manufactured 3D-Nanoprobe is very robust due to its compliant flexure hinge structure.

After calibration, the 3D-Nanoprobe’s performance was tested regarding its probing repeatability and classic measurement tasks in dimensional nanometrology. The probing repeatability for 100 points was about 0.18 nm, 0.31 nm, and 0.83 nm for *x*, *y*, and *z*, respectively. The 3D-Nanoprobe was tested on two orthogonally mounted IVPS100-PTB reference samples [22]. These reference samples contain lines with parallel sidewalls, sidewall angles of 90° and sharp corners. The CD value of those IVPS100-PTB samples was measured regarding its repeatability, stability, and pitch as well as its line edge roughness and the linewidth roughness. The repeatability measurements of the one so-called S5 line on the IVPS100-PTB samples showed a decrease of −0.33 pm and −5.4 pm per line with 240 points, in the *x*- and *y*-direction, respectively. The sidewall angle with about 89.5° and about 90.0° in the *x*-direction for left and right sidewall, respectively, shows good agreement with the IVPS100-PTB nominal sidewall angle of 90°. The sidewall angle in the *y*-direction was biased by the incident angle of the 3D-Nanoprobe and showed about 89.8° and 87.6° for the left and right sidewall, respectively. Unfortunately, the current tests were performed with 3D-Nanoprobes without tilt compensated CD tips. For this reason, the right sidewall in the *y*-direction could not be probed completely because the CD tip had only a small overhang of a few nanometers, and the CD-tip shaft came into contact with the sample. This probing condition seems to have an influence on the long-term stability measurement of the CD-value in the *y*-direction. It also seems to affect the higher total changes of 1.2 nm which occurred compared to the orthogonal *x*-direction with an overall decrease in the CD value of about 0.7 nm and a trend of less than −0.3 pm per line.

The pitch measurement on the IVPS100-PTB in the *x*-direction showed very good consistency with a very small increase of 6.6 pm per measurement. The pitch measurement on the IVPS100-PTB in *y*-direction is biased by the tilted CD-tip and shows a bigger change compared to the *x*-direction of −127.8 pm per measurement. The left sidewall of the pitch measurement was used to evaluate one-side probing. The distance of the left sidewall of the line structures, S4 and S5, showed an increase of 1.6 pm and a decrease of −126.9 pm in *x*- and *y*-direction, respectively. This indicates a tip abrasion/wear process on one side of the CD tip during the VP-line scan for each individual alternating measurement in the *y*-direction.

Lastly, also the line edge roughness and linewidth roughness were also tested along a trace of 2 µm. The IVPS100-PTB for the *x*-direction showed a line edge roughness of S_q_ = 0.75 nm and a corresponding linewidth roughness with a S_q_ = 1.49 nm. The IVPS100-PTB for *y*-direction showed a S_q_ line edge roughness of 0.44 nm and the corresponding S_q_ line-width roughness of 1.46 nm. Two repeated measurements of the left line edge in the *x*- and *y*-direction demonstrate the very good repeatability of the sidewall profiles.

To conclude, the novel concept of the 3D-Nanoprobe was proved though experiments, and the proposed model of the detection system—which was used to design the true 3D-AFM head—shows good agreement with the experiments.

Looking forwards, the 3D-Nanoprobe will also be manufactured with smaller CD-tip sizes to show the clear benefits of the compliant flexure hinge structure, and CD probes with pre-tilted DLC CD-tips will be used. Moreover, with this new working 3D-Nanoprobe the next generation of 3D-tip control will be developed to improve tip–sample interaction and to further reduced tip slipping and tip wear. As a result of that, the 3D-Nanoprobe might also be used as a force transducer to investigate material properties in the future.

## Figures and Tables

**Table 1:** Stiffness values of three manufactured 3D-Nanoprobes obtained by calibration and finite element method (FEM) simulations.

3D-Nanoprobe	Measured Stiffness in N/m (*k* = 2)	FEM Modelled Stiffness in N/m
A ^1^	$k_{x}=1.722\;\pm \;0.083$ $k_{y}=1.511\;\pm \;0.034$ $k_{z}=1.64\;\pm \;0.16$	$k_{x}=2.13$ $k_{y}=1.98\;$ $k_{z}=2.45$
B ^2^	$k_{x}=2.016\;\pm \;0.024$ $k_{y}=2.036\;\pm \;0.070$ $k_{z}=2.311\;\pm \;0.089$	$k_{x}=2.11\;$ $k_{y}=2.23\;$ $k_{z}=2.72$
C ^3^	$k_{x}=2.719\;\pm \;0.067$ $k_{y}=2.461\;\pm \;0.065$ $k_{z}=1.198\;\pm \;0.099$	$k_{x}=2.22\;$ $k_{y}=2.42\;$ $k_{z}=2.32$

^1:^*φ tiltA* = 8.3°, ^2:^ *φ tiltB* = 8.5°, ^3:^ *φ tiltC* = 6.8°; with *φ Setup* = 8°.

## Data Availability

The data is available upon request.
